# Supplementary material for: Misleading graphs in context: Less misleading than expected
Source: PLoS One. 2022 Jun 15;17(6):e0265823. doi: 10.1371/journal.pone.0265823 (PMC9200168; doi:10.1371/journal.pone.0265823)
Supplement: S1 Appendix — (DOCX) [file pone.0265823.s002.docx]

# S1 Appendix: The Questionnaire

In the survey below, the following coding is used as abbreviation for the four distinct surveys:

1. Normal graph; endangered context
2. Shifted graph; endangered context
3. Normal graph; ecosystem context
4. Shifted graph; ecosystem context

## Introduction

**Version A/B**

An ‘Endangered species’ is a type of animal or plant that might stop existing because there are only a few of that type alive. Have you heard of this before?

*No, Maybe, Yes*

If endangered animals are not protected, they go extinct. That means that there will never be an animal of this species again. Have you heard of this before?

*No, Maybe, Yes*

How do you feel about endangered animals?

*Do not care at all, mostly do not care, neutral, concerned, very concerned*

**Version C/D**

The word ‘eco-system’ means: all the living things in an area and the way that they affect each other and the environment. Have you heard of this term before?

*No, Maybe, Yes*

An eco-system nay become unbalanced. This can be caused by natural disasters such as floods and volcanic eruptions. It can also be caused by humans through, for example deforestation and pollution. Another cause is the increase of the amount of certain non-native animals, as they might hunt or eat to much, leaving little food for other animals.

Animals and plants find it harder to survive in an imbalanced eco-system. Have you heard of this before?

*No, Maybe, Yes*

How do you feel about imbalanced eco-systems?

*Do not care at all, mostly do not care, neutral, concerned, very concerned*

## The ‘Bluebeak’

**Version A/B**

The ‘Bluebeak’ is an endangered animal. The small predator bird lives in Denmark. In recent years the number of Bluebeaks in recent years the number of Bleubeaks in Denmark has increased. This can be seen in the following figure

*(One of the two panels of Figure 1 is shown here)*

What is your judgement on the increase of the number of Bluebeaks?

*Very bad, bad, neutral, good, very good*

**Version C/D**

The ‘Bluebeak’ is a small predator bird. It is non-native to Denmark and disrupts the eco-system. In recent years the number of Bluebeaks in Denmark has increased. The can be seen in the following figure.

*(One of the two panels of Figure 1 is shown here)*

What is your judgement on the increase of the number of Bluebeaks?

*Very bad, bad, neutral, good, very good*

## Estimation

With the graphs in your memory, can you guess how much the amount of Bluebeaks has increased from 2014 to 2019?

My guess (in percentages): ___

## Short graph literacy test

*Here, the Short graph literacy test from Okan et al. (2019) is reproduced.*

## Measurement of knowledge that graphs can be misleading

Think about graphs that you might have seen in different contexts, such as graphs presenting data for different financial, nutritional, or political options and trends. Do you think that sometimes they are designed in a way that….

Makes some options look better or worse than they really are (e.g., by making differences in the data presented look larger or smaller)?

*Yes, no, I do not know*

Directs attention to a particular option or aspects of that option (e.g., by directing attention to specific values in the data)?

*Yes, no, I do not know*

Makes trends look more positive or negative than they really are (e.g., by distorting or misrepresenting the trends in the data)?

*Yes, no, I do not know*

## Demographic questions

What is your age?

*18-25, 26-30, 31-35, 36-45, 46-55, 56-65, 66+, I would rather not answer*

What is your gender?

*Male, Female, Other, I would rather not answer*

What is the highest level of education you have completed?

*No formal qualifications, High school diploma/ A-levels, Secondary education (e.g., GED/GCSE), Technical/community college, Undergraduate degree (BA/BSc/other), Graduate degree (MA/MSc/Mphil/other), Doctorate degree (PhD/other), do not know/not applicable, I would rather not answer*
