# Supplementary material for: Misleading graphs in context: Less misleading than expected
Source: PLoS One. 2022 Jun 15;17(6):e0265823. doi: 10.1371/journal.pone.0265823 (PMC9200168; doi:10.1371/journal.pone.0265823)
Supplement: Attachment — Submitted filename: reg-tab.pdf [file pone.0265823.s003.pdf]

	Judgement		
Predictors	*Estimates*	*CI*	*p*
(Intercept)	2.21	1.48 – 2.95	**<0.001**
Story [Endangered]	1.88	1.67 – 2.09	**<0.001**
Graph [shifted]	0.25	-0.10 – 0.59	0.157
c SGL	-0.16	-0.63 – 0.31	0.492
Gender	0.02	-0.31 – 0.35	0.918
f educ [3]	-0.31	-1.01 – 0.39	0.381
f educ [4]	-0.07	-0.76 – 0.61	0.839
f educ [5]	0.01	-0.72 – 0.74	0.976
f educ [6]	-0.32	-1.21 – 0.58	0.488
f educ [7]	-0.48	-1.63 – 0.67	0.413
f educ [9]	-0.02	-0.73 – 0.70	0.960
f age [3]	0.14	-0.27 – 0.54	0.502
f age [4]	0.10	-0.31 – 0.51	0.636
f age [5]	0.02	-0.38 – 0.41	0.932
f age [6]	0.05	-0.32 – 0.43	0.775
f age [7]	0.07	-0.35 – 0.48	0.745
f age [8]	0.07	-0.35 – 0.48	0.749
Graph [shifted] * c SGL	0.47	-0.20 – 1.15	0.170
Graph [shifted] * Gender	-0.07	-0.56 – 0.42	0.779
c SGL * Gender	0.18	-0.46 – 0.81	0.587
(Graph [shifted] * c SGL) * Gender	-0.94	-1.85 – -0.03	**0.042**
Observations	247		
R^2 / R^2 adjusted	0.606 / 0.572		
