# Supplementary material for: Misleading graphs in context: Less misleading than expected
Source: PLoS One. 2022 Jun 15;17(6):e0265823. doi: 10.1371/journal.pone.0265823 (PMC9200168; doi:10.1371/journal.pone.0265823)
Supplement: Attachment — Submitted filename: Rplot05.pdf [file pone.0265823.s004.pdf]

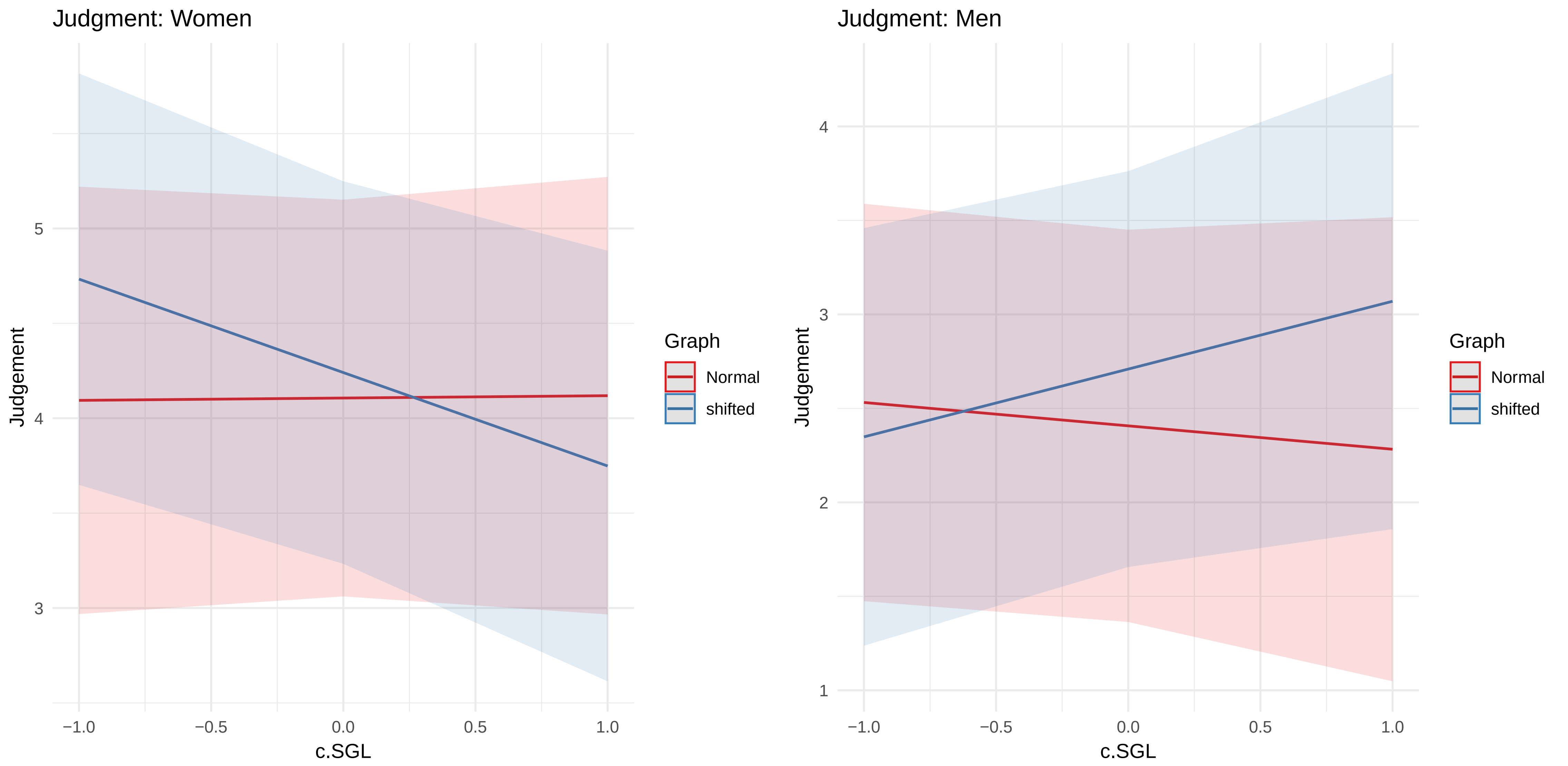
Judgment: Women
Judgement
5
4
3
−1.0
−0.5
0.0
0.5
1.0
c.SGL
Graph
Normal
shifted
Judgment: Men
Judgement
4
3
2
1
−1.0
−0.5
0.0
0.5
1.0
c.SGL
Graph
Normal
shifted
